# Death of Dr. Peck, Associate Editor

**Published:** 1915-07

**Authors:** William J. Schuyler

**Affiliations:** Secretary


### Death of Dr. Peck, Associate Editor.

Dr. Fayette H. Peck, New York University Medical College 1881, died at his home in Utica, May 24, aged 59. He was affiliated with the Oneida County, State and national organizations and was visiting surgeon to St. Luke's Hospital.

The death of Dr. Fayette H. Peck was a great shock to the community in which he lived, and a great loss to the profession of which he was such an honored and beloved member. His death was unexpected and sudden from apoplexy.

Dr. Peck's career had brought him in contact with many of the best men in the State of New York, and he was more than a local figure. In the first place, he had a splendid physique, was well-educated, being a member of the same class and fraternity as the Hon. James S. Sherman, Vice-President of the United States, recently deceased, and he was his physician and friend for many years.

His railroad appointment as surgeon gave the opportunity to show the energy and resourcefulness of his nature, and he was well-trained and alert, with a heart that was sympathetic and strong. All over the State of New York he will be remembered by physicians as a charming man to meet and to know, and in central New York he will be remembered as a strong and able ally of every good cause, and an earnest worker for the best ideals of our profession.

He was the senior surgeon of St. Luke's Hospital, had many local appointments, and was a member of various societies, both state and national, and was a factor in the medical affairs of the state that could be reckoned always on the right side of questions which his intelligence and judgment endorsed. Following is a short local tribute by the physicians of St. Luke's Hospital which are expressions of local feeling regarding the loss of this really valuable member of our profession.

Resolutions of Respect.

At a formal meeting of St. Luke's Hospital medical staff, jointly held with the Clinical Society of the hospital, the following resolution was adopted and the secretary was directed to send copies to the family and to the newspapers for publication:

"The death of Dr. Peck has not only removed a strong man from our midst, but has touched us with great sorrow. The doctor has for many years been enthusiastic in all those professional activities, in which we are most deeply interested; more than this, his charming personality has endeared him to us all. He was instinctively fair and honorable, cheerful and always a gentleman. His ability was recognized by the entire profession. He started right in life, his education did not mar his admirable personal traits, and his professional training added culture to his fine character. We greatly miss him, and will sincerely mourn our great loss."

By WILLIAM J. SCHUYLER,

Secretary.

